# Design and experiment of tea winnowing parameter control system based on YOLO-AE

**DOI:** 10.3389/fpls.2025.1721083

**Published:** 2026-01-21

**Authors:** Kun Luo, Yangyang Huang, Xuechen Zhang, Zhiqiang Li, Jie Xiong, Dongsheng Wang, Rongchao Liu, Liang Tao, Yujie Wang

**Affiliations:** 1 School of Mechanical Engineering, Tongling University, Tongling, China; 2 School of Computer and Software Engineering, Anhui Institute of Information Technology, Wuhu, China; 3 College of Biosystems Engineering and Food Science, Zhejiang University, Hangzhou, China

**Keywords:** deep learning, tea pneumatic separation, real-time control, YOLO-AE, tea processing

## Abstract

Tea winnowing is a key process in tea processing. At present, tea winnowing parameters are adjusted by manual observation of tea leaves. This results in the uncertainty of winnowing quality. In this work, we propose a new tea winnowing method based on deep learning for the characteristics of white tea. Firstly, the YOLOv11 model is improved by introducing ACmix and EUCB. The recognition accuracy of the improved YOLO-AE model is improved by 2.1%, and the detection time is shortened by 40%, which significantly improves the detection performance and shortens the inference time. The region segmentation and convolution neural network algorithm are used to distinguish the proportion parameters of each grade in tea in real time, and the accurate wind selection parameters are obtained by combining the winnowing theory. The recognition accuracy of the verification set of the recognition model attains 94%. The MAP (0.5:0.95) is 0.93. A test on the tea winnowing parameter control test bench reveals that the identification accuracy of tea materials with different proportions is consistent. Additionally, the difference between the two batches of high-quality white tea is less than 3%. The winnowing scheme proposed in this study can provide the basic theory and technical support for the design of tea precision winnowing equipment.

## 1 Introduction

Tea is one of the important economic crops in China. With the improvement in living standards, the demand for high-quality tea has increased (Liu, 2019). Tea production is considerable in summer and autumn. However, because tea is mixed with more impurities such as tea stems and broken leaves, the price is low. The impurities in summer and autumn tea can be removed by pneumatic separation. The price of tea after removing impurities is 3–5 times that of the raw materials (Gan et al., 2018). However, the pneumatic separation parameters required for different types of tea or different batches of the same type of tea vary. If the pneumatic separation parameters are not adjusted accurately in a timely manner, the quality of pneumatic separation would decrease rapidly.

At present, the tea sorted by a color sorter displays high precision. However, the efficiency of sorting cannot satisfy the requirements of mass production of tea, and the price is high. The sorting of large quantities of tea relies mainly on pneumatic separation equipment. However, the current structural parameters of tea pneumatic separation rely on manual experience design. Workers cannot correct the air separation parameters in a timely manner according to the different raw materials of tea. This results in inconsistent quality of different batches of tea.

A substantial amount of research has been conducted on tea classification worldwide. The tea sample information was obtained by spectroscopy. Then, the SVM model was optimized by particle swarm optimization, artificial bee colony, genetic algorithm, and other algorithms. Finally, an accurate classification of tea was realized (Mishra et al., 2019; Hussain et al., 2021; Mingyin et al., 2021; Yuhan et al., 2022). At the same time, some scholars use YOLO model to identify tea buds, which has a good recognition effect in complex background (Lyu et al., 2021; Xu et al., 2022; Cheng et al., 2023; Dong et al., 2024). The physical parameters of tea, such as area, perimeter, and diameter, can be used as indexes to distinguish the grade of tea by a machine vision system (Rahman et al., 2021). Jiang et al. used the symmetry detection algorithm to detect the edge of tea images. This further improved the efficiency and accuracy of tea classification (Jiang and Chen, 2020). Song et al. obtained the histogram of tea shape features. Through various machine learning model recognition techniques, it was determined that the model based on shape feature histogram is the best (Yan et al., 2018). Bakhshipour et al. extracted the color and texture features of four types of black tea. They observed that the neural network constructed by correlation feature selection had the best classification effect (Bakshipour et al., 2018). Sun et al. collected five types of hyperspectral images of green tea and established a support vector machine model for green tea recognition. The classification accuracy rate attained 96% (Sun et al., 2018). The above research results reveal that the deep learning algorithm has a good effect on tea recognition and classification. However, the experiment determined that the existing model has a low accuracy in identifying samples with similar tea. Therefore, it is urgent to develop an accurate identification and classification method for similar variations in tea parameters to prevent the adverse impact on the windward side and weight assessment in the subsequent classification and statistics.Zhang et al. studied the flow field (different wind speed distributions) of tea particles with different weights at different air duct outlets. The influence of drift trajectory, horizontal drift law, and wind direction angle on the quality of tea air separation (Zhang et al., 2013). Zhong et al. used the method of simulation analysis to study the trajectory of tea particles under different wind speed distributions in a tea air separator. When the wind speed is distributed according to upper small and lower large, the wind selection effect of tea particles of different quality is better. The impurity rate of each tea outlet can be reduced by increasing the spoiler (Zhing et al., 2013). Wu et al. designed a double-layer tea air separator and established an automatic control system based on a programmable logic controller. The separation baffle of a tea air separator was adjusted electrically using a frequency converter. Then, the optimum process parameters were obtained by an orthogonal test (Zhengmin et al., 2022). Luo et al. defined the simulation environment of tea material, flow field, and wind field wall by fluid-solid coupling. Through the simulation test, the wind selection parameters of tea were determined accurately, and the accuracy of tea wind selection was improved (Luo et al., 2022). The above researchers mainly simulated and analyzed the air separator through theoretical analysis and fluid dynamics methods to improve the structure of the pneumatic separation. The aim was to improve the air separation effect. Few studies have been conducted on how to configure the parameters of the air separator according to different tea types and impurity rates to realize intelligent air separation. Considering the practical application, the following problems persist: (1) The current research is focused on the slower environment, which cannot satisfy the real-time online recognition of the air separator. (2) The proportion of each grade of tea cannot be analyzed statistically in real time. (3) At present, there is no application of deep learning recognition combined with wind selection.

To address the demand for high efficiency and high precision for large quantities of tea grading, this study combined the previous research foundation of the fluid–solid coupling tea wind separation theory. A sorting scheme combining machine vision and air separator was proposed. First, the identification model of different components of tea was established, and the grade proportion of export tea was analyzed online in real time. Combined with the adjustment model of winnowing parameters, the accurate winnowing parameters required for current tea were determined. Then, the air separation parameters of the air separator were adjusted in real time so that different tea materials can maintain the best separation parameters in real time. The specific summary is as follows. Because the tea in this study is a small target, and the characteristics of different types of tea are similar, the recognition accuracy of the model is improved by further optimizing YOLOV11, and finally the accurate and efficient winnowing is realized.

## 2 Materials and methods

### 2.1 Dry tea material parameters

**Figure 1:**
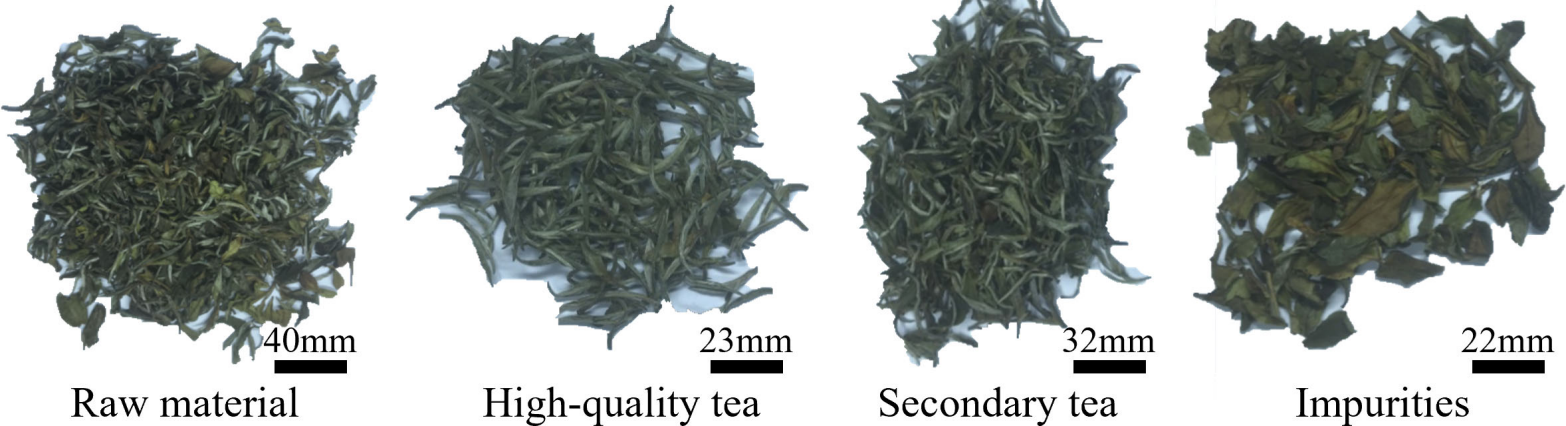
Composition of mixed tea.

The experimental material used in this study was white tea raw material. The preliminary processing technology of white tea involves picking, withering (withering + screening), drying, and other processes. After the preliminary processing of tea. White tea products are formed through fine processing (picking, stacking, blending, stacking, redrying, and packing). After the initial processing of the white tea, the main impurities in the mixed material are yellow flakes and broken powder. Fine processing can remove most of the yellow flakes and debris impurities by air separation. The special white tea is selected from the secondary white tea by sorting. The main components of the test materials selected in this study were high-quality white tea (all single buds, used to make white silver needles), secondary white tea (mainly a bud and a leaf, a bud and two leaves, used to make white peony), and yellow flake impurities (**Figure 1**).

### 2.2 Winnowing platform based on deep learning

**Figure 2:**
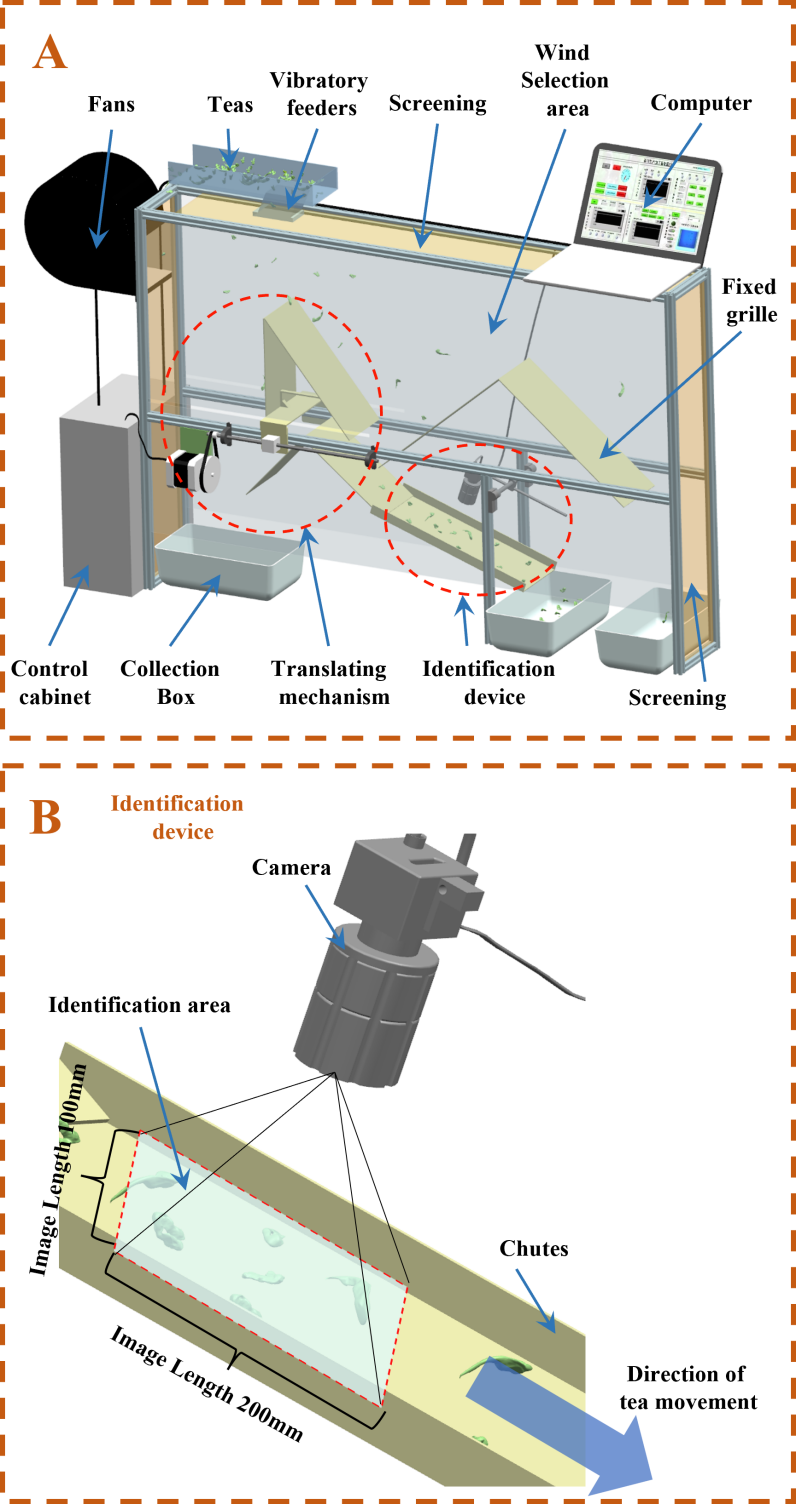
Winnowing parameters real-time adjustment test bench [**(A)** whole machine diagram; **(B)** recognition system structure diagram].

For the fine processing of white tea. The problem of poor air separation effect caused by inaccurate manual setting of air separator parameters. In this study, a real-time control experimental platform for tea selection parameters based on deep learning was designed. It is illustrated in **Figure 2**. The proportion of tea components at the outlet position is calculated in real time by the recognition model. Then, the actuator is controlled to adjust the air separation parameters in real time. Its overall structure is shown in **Figure 2A**. The winnowing platform is mainly composed of a tea recognition system, separator position control system, and winnowing mechanism. The tea is entered uniformly into the internal of the air separator through the vibrating feeder. The fan produces air flow into the fan horizontally. The tea drifts to the right under the action of the air flow inside the wind separator. Different types of materials in the tea material fall in different grilles below. Tea slides through the identification area through the slideway. The camera directly above the slideway captures the image. The tea identification system to determine the impurities of tea and related parameters to identify the current batch of tea require winnowing parameters. The specific structure is shown in **Figure 2B**. The control system adjusts the wind speed, grille position, and feeding amount of the fan to achieve the best separation quality.

### 2.3 Real-time recognition model of white tea material proportion

#### 2.3.1 White tea sample data collection

For more accurate results of white tea material category identification and analysis, the captured tea image needs to be clear and the background contrast needs to be high. Images with clear contours are convenient for manual labeling and for machine learning and recognition. The test bench selects the strip LED light source as the light source of this test. The image acquisition device is an industrial camera (acA1920-150uc, Germany). The size of the shooting area is 200 × 100 mm. The shooting distance is 220 mm. The discharge port adopts a blue background plate. Blue is more conducive to the shooting of tea images, reducing the exposure of images, preventing shadows in images, and improving the quality of images than other color background plates. Through the image acquisition platform, a total of 1095 sample images were collected in this experiment. These include 426 high-quality white tea images, 462 secondary white tea samples, and 207 yellow chips (yellow chips, broken leaves, and tea chips) samples. To improve the diversity of datasets to adapt to more complex environments, this study increased the number of images in the dataset by color adjustment and mirror flipping of images. After amplifying 1095 datasets, 5756 images were obtained as the final dataset. Among the 5756 samples, the training, validation, and test sets accounted for 70%, 20%, and 10%, respectively.

#### 2.3.2 White tea category recognition model

Faster R-CNN, SSD, and YOLO are the most widely used object detection models. The defects of YOLOV11 model in identifying small tea targets are improved.In this study, four models were used to train the white tea grade recognition model. First, the dataset constructed in-house in this study was used to input the three models to generate the corresponding level recognition model. Then, the results were compared and analyzed. Finally, the optimal model was selected to construct the white tea grade recognition model in this study.

**Figure 3:**
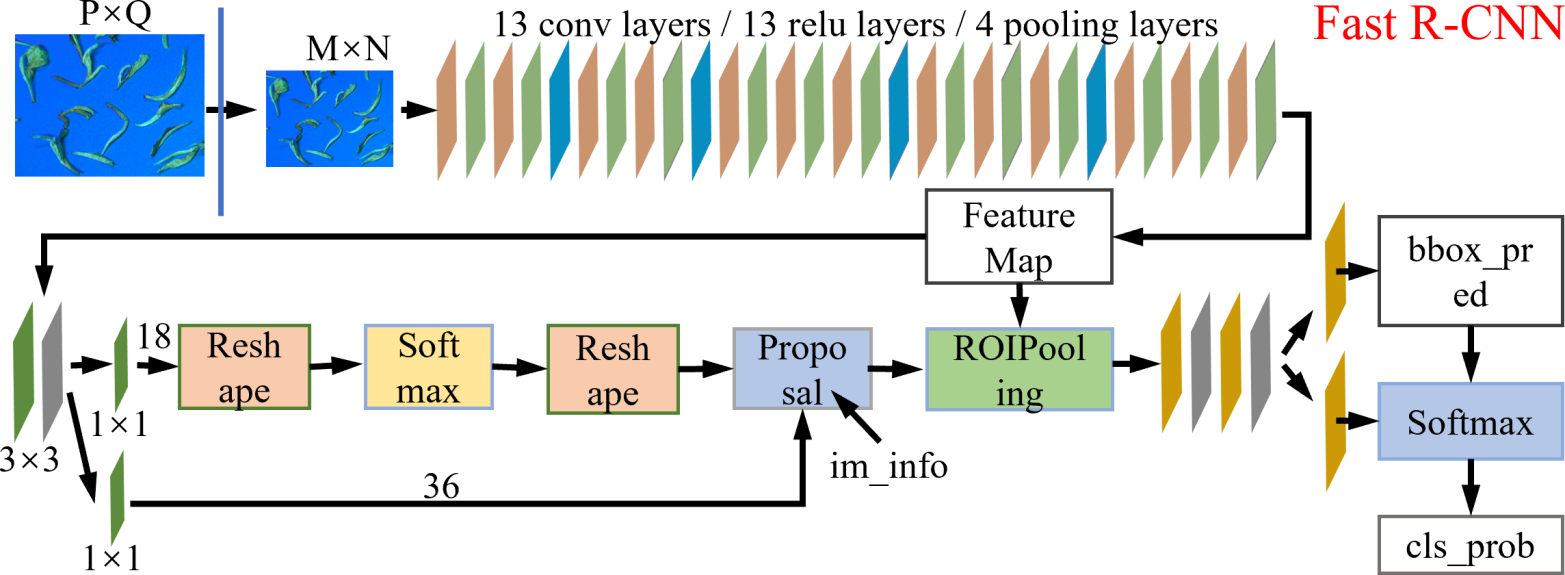
Faster R-CNN algorithm model.

The Faster R-CNN model is based on the R-CNN and Fast R-CNN network structure. The model mainly combines feature extraction, candidate box extraction, bounding box regression, and classification. This considerably improves the detection speed. The network structure is shown in **Figure 3**.

**Figure 4:**
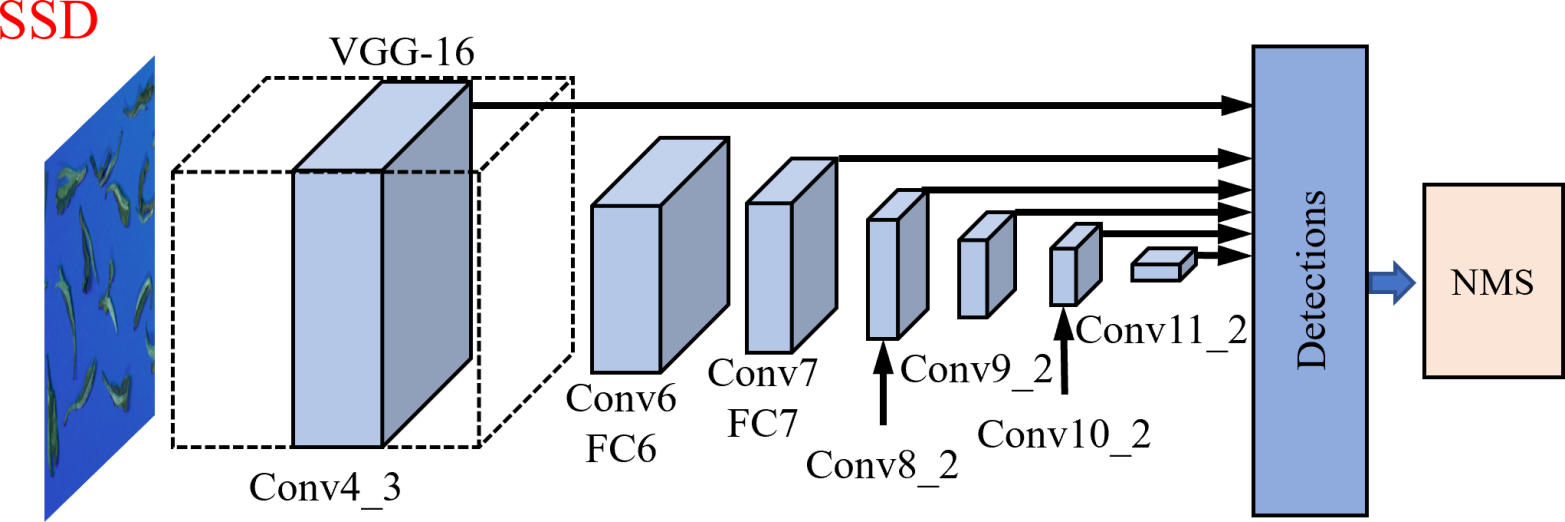
VGG-16 and SSD model network structure.

The backbone network used by the SSD model is VGG-16, as shown in **Figure 4**. It is the basic framework of the VGG-16 model. The feature extraction network of the SSD network model is optimized for VGG-16.

**Figure 5:**
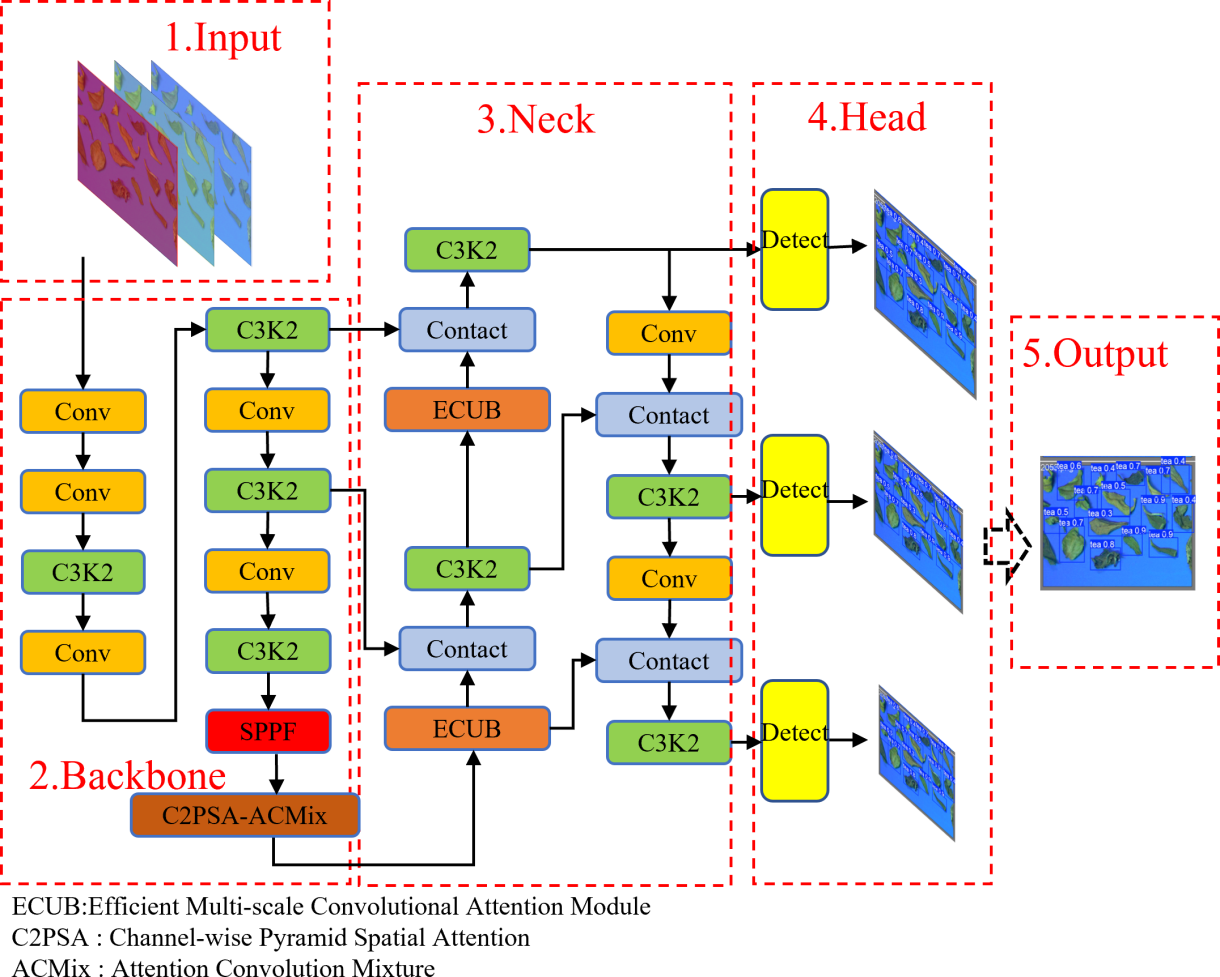
YOLO-AE model network structure.

The tea that needs to be identified in this study is a small target, and it needs a higher speed when it is identified, and it also needs to be lightweight when deployed on the device. Compared with YOLOV5 and YOLOV8, YOLOV11 introduces C3K2/REP module to reduce inference delay. Integrated attention mechanism to improve global perception. At the same time, YOLOV11 is more suitable for the pursuit of accuracy and real-time scenes. Therefore, this study introduces a C2PSA-ACmix hybrid module at the end of the backbone network of YOLOV11, which combines the advantages of self-attention mechanism and convolution operation. ACmix first uses 1x1 convolution to project the input feature map to generate a set of intermediate features, and then reuses and aggregates these intermediate features according to different paradigms. Aiming at the difficulty of detecting dense small targets such as tea, a feature enhancement module (EUCB) is introduced at the key position of the head network. The workflow includes: 1.Bilinear upsampling (2 ×) expands the spatial resolution of the feature map. The DWC-BN-ReLU sequence achieves efficient feature nonlinear transformation. 3.1×1convolution completes channel dimension simplification to ensure compatibility with downstream networks. The self-attention module gives the model long-range dependent modeling ability, while the depthwise separable convolution (DWC) effectively captures local texture details. The combination of the two significantly improves the robustness of feature representation. The network structure is shown in **Figure 5**.

The analysis of the ACmix module has two phases. In the first stage, three 1 × 1 convolution projections are performed on the input feature map. In the second stage, the outputs of the two paths of Convolution aggregation and self-attention aggregation are added, and the intensity is controlled by two learnable scalars (Pan et al., 2022). The expression is shown in Equation 1.

(1) $$F_{out}=\alpha F_{att}+\beta F_{conv}$$

The ECUB module is based on the attention mechanism. The core idea is to extract features by deep separable convolution DWC (.) Then the batch normalization operation BN (.) is performed to stabilize the training process. The nonlinear transformation is introduced by the ReLU (.) activation function. Finally, 1 × 1 convolution is used to compress the number of channels in the feature map. Make it match with the input requirements of the next stage (Rahman et al., 2024). The expression is shown in the Equation 2.

(2) EUCB(x)=C1×1(ReLU(BN(DWC(Up(x)))))

#### 2.3.3 Identify model evaluation indicators

The computer used for model training is configured as a CPU processor (12th Gen Intel (R) Core (TM) i7-12700H 2.70 GHz). The graphics card is NVIDIA RTX3060 GPU, and the memory is of 16 GB. The operating system running the model is Windows 11 Professional 64 bit. The software tool is PyCharm 2021.3.2. The experiment was implemented in the Pytorch framework. To evaluate the effect of the model in identifying the tea shoots, this study used the recognition accuracy, recall rate, and map as the evaluation indexes to evaluate the training recognition model. The specific calculation indexes are shown in Equations 3–5.

(3) $$P_{C}=\frac{TP}{TP+FP}$$

(4) $$R_{C}=\frac{TP}{TP+FN}$$

(5) $$F_{C}=\frac{2P_{C}R_{C}}{P_{C}+R_{C}}$$

Among them, TP is the number of correctly identified white tea categories, FP is the number of misidentified white tea categories, and FN is the number of unidentified white tea categories. For the tea target detection task in this study, the definition of TP and FP is based on the following two criteria: (1). The category of the prediction box must be consistent with the category of the real target. (2). IOU threshold: The IOU value between the prediction box and the real target box is greater than 0.5. If the prediction box satisfies the above two conditions, it is regarded as TP. If the prediction box does not meet any of the above conditions, it is considered FP.The larger the values of PC, RC and FC, the higher the accuracy of the model to identify the white tea category. The time required for the tea to enter the camera field-of-view to obtain the type information of the white tea category by the recognition model also reflects the superiority of the model from the side. The shorter the average processing time, the better is the model.

### 2.4 Statistical analysis algorithm of white tea material category

**Figure 6:**
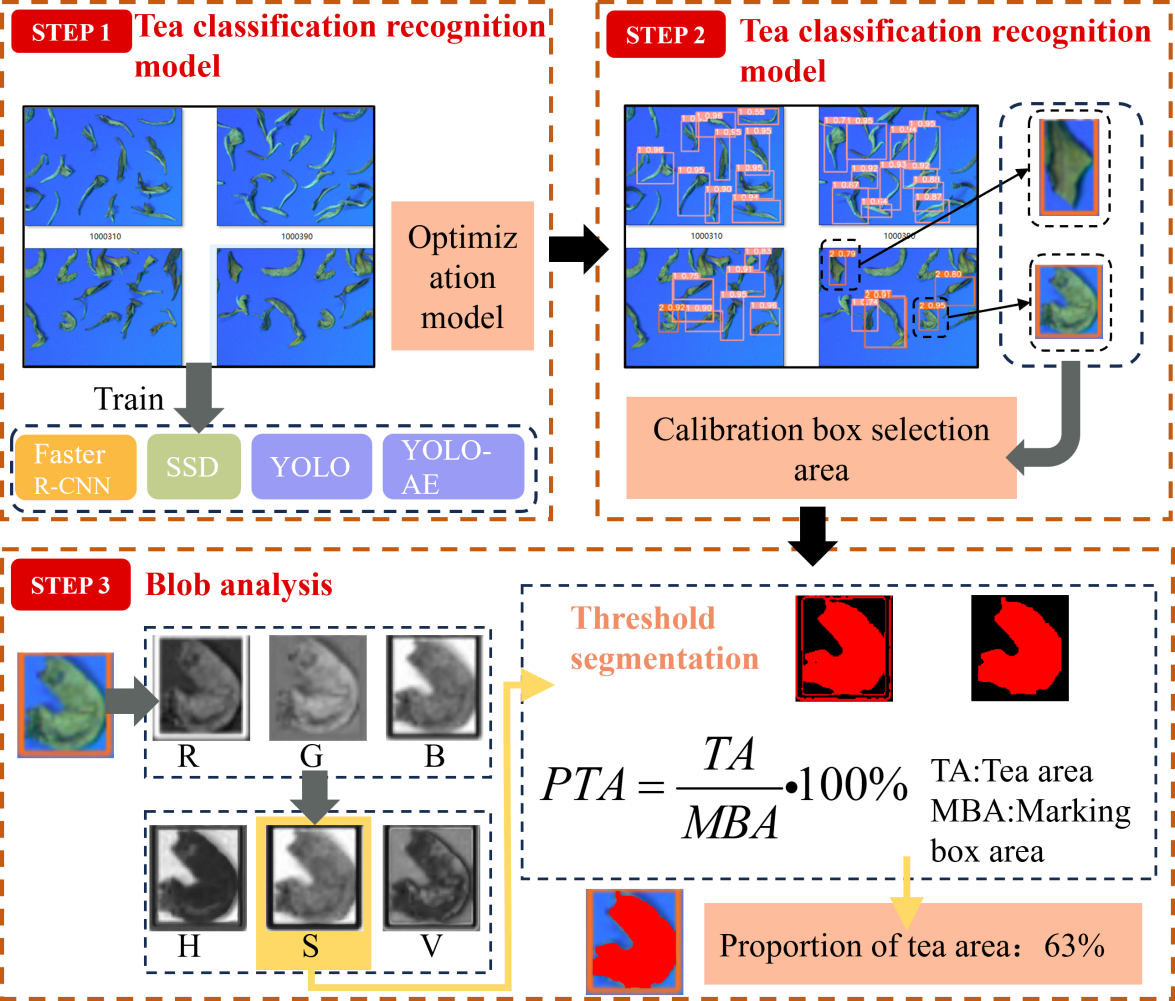
Tea category statistical analysis algorithm.

When the white tea recognition model detects different types of tea materials, the model also needs to count the number and area proportion of different tea material types. The unit number and area of tea determine the setting of wind selection parameters. Therefore, in this study, the number and area of different materials were determined by region segmentation, and the volume of different materials was estimated by combining the shape characteristics of different materials. The weight was determined based on a statistical analysis of the density of different types of materials. The material statistical analysis process is shown in **Figure 6**.

The first step was to use the camera to obtain the tea photographs at the outlet of the tea air separator. Tea recognition tests were performed using the trained faster R-CNN, SSD, YOLO, and YOLO-AE models. Finally, the best recognition model was selected for the tea recognition experiment. The second step was to use the marker box to indicate the tea types identified by each model. Then, the region segmentation algorithm was used to extract the tea image in the marker box region (Wang et al., 2024; Chen et al., 2025). The third step was to extract the images of the R, G, B, H, S, and V channels from the segmented tea images. The channel with the highest contrast was selected for the area ratio calculation. Then, the image threshold segmentation of the tea S channel was extracted to the tea area. The areas of the tea area and marker box were obtained by area calculation. The ratio of the two was used as the ratio of the tea area.

Ten tea materials were selected randomly. The density and thickness parameters of different tea types were analyzed statistically. The specific results are shown in **Table 1**. The distance and area utilized by the camera are fixed. Therefore, the proportion of each tea can be determined by counting the number of various teas and then calculating the total proportion of the area occupied by the images. By counting the density and thickness of different types of tea, the weight can be calculated according to the measured tea area. By calculating the tea area and weight, the adjustment of the wind selection parameters was determined.

**Table 1:** Table of physical parameters of tea materials.

Tea style	Density/(ρ)	Compositional ratio/%)	Thickness
Tea stem	455	5.6	0.7–1.5
High quality tea	232	37.4	1.1–1.9
Tight leaf	250	21.2	2.1–3.3
Tatter leaf		35.1	0.7–1.2
Crushed foam		3.1	

### 2.5 Winnowing parameter adjustment mechanism

**Figure 7:**
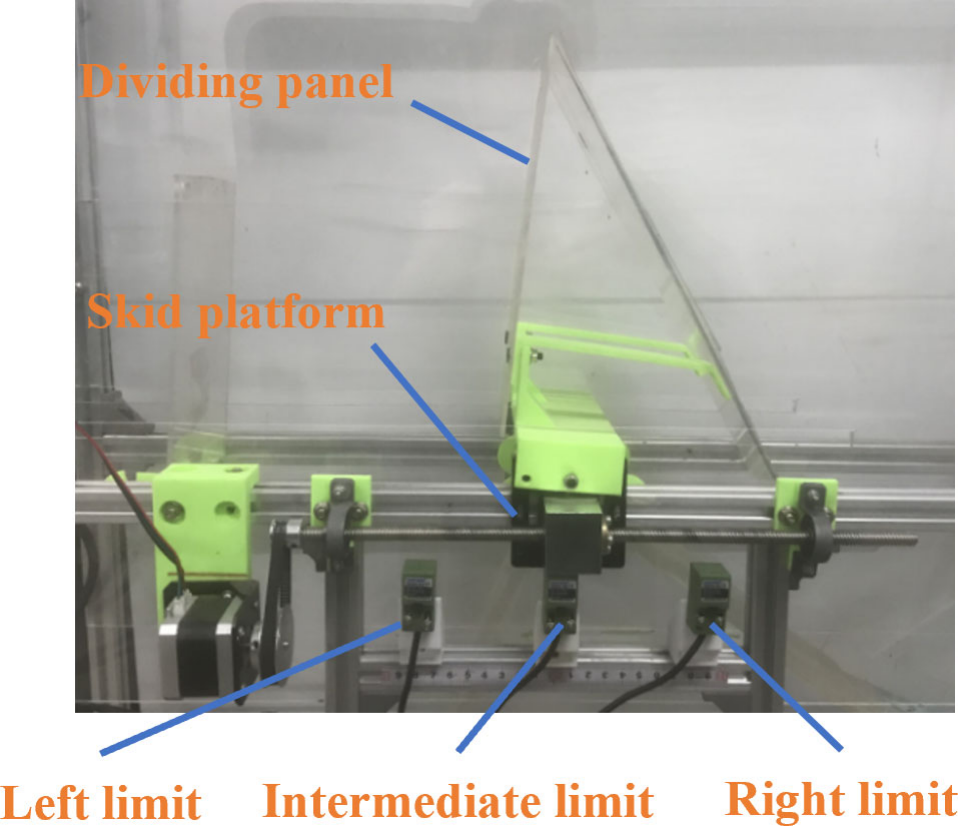
Partition plate regulating system structure diagram.

After the identification system determines the proportion of different types of tea materials. According to the previous research results of wind field simulation, the size of the blanking port is adjusted by adjusting the left and right position parameters of the partition plate. The moving mechanism of the dividing plate is shown in **Figure 7**. The partition plate is fixed on the slider. The slider moves toward the left and right through the rotation of the screw. One end of the screw is connected with the stepping motor. The positions of the partition plate are left, right, and origin restriction. The reference point of the dividing plate is the origin when adjusting the position each time. The left and right limits ensure that the partition plate does not exceed the limit position.

**Figure 8:**
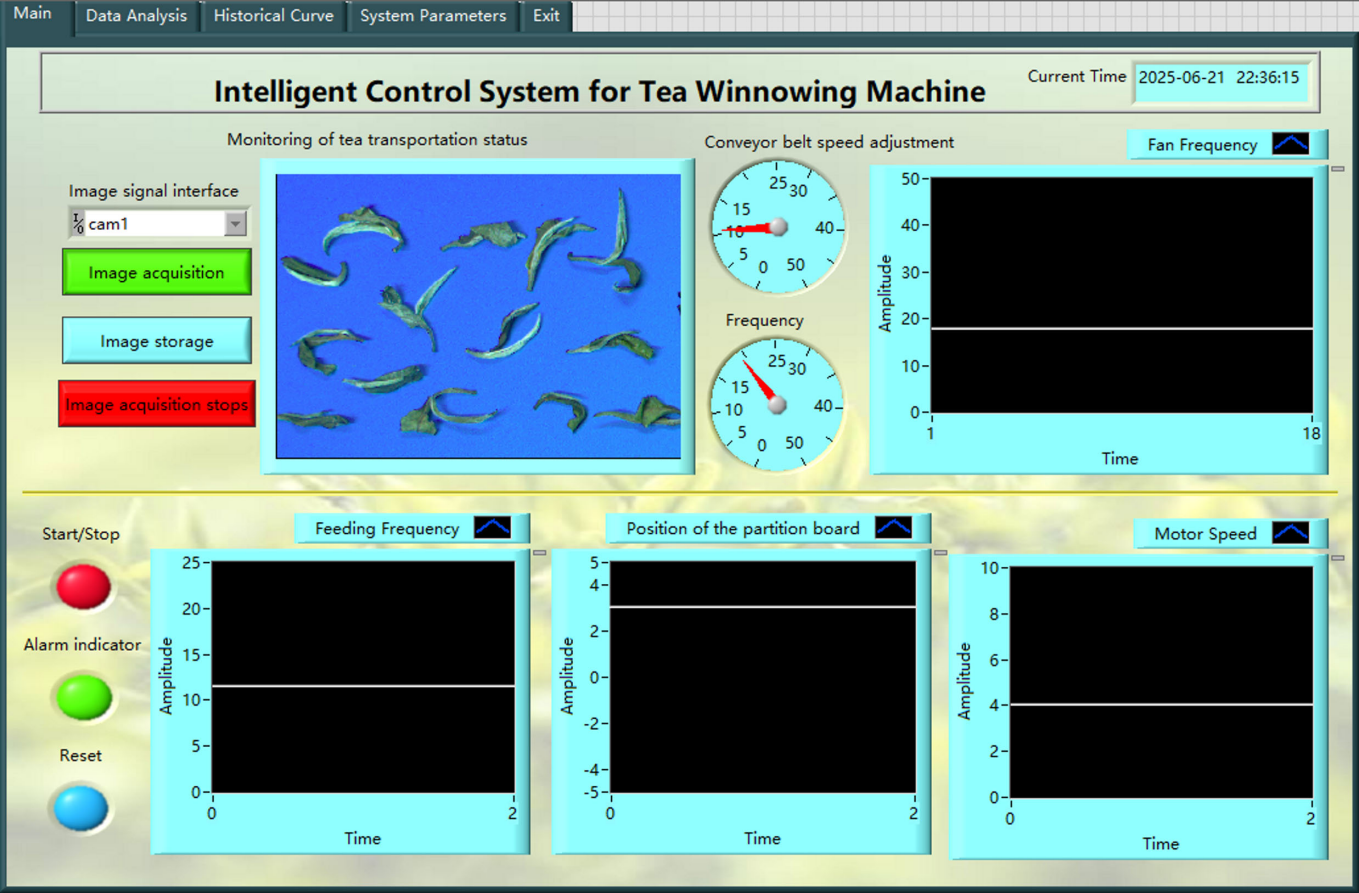
Winnowing test bench control system.

The intelligent control system of tea air separator is shown in **Figure 8**. The host computer control system integrates related information such as the fan frequency, feeder frequency, baffle position, current wind speed, and tea image at the discharge port. The parameters in the process of air separation can be observed and recorded in real time through the upper computer control system. Simultaneously, the control system can be controlled manually and automatically.

## 3 Results and discussion

### 3.1 Model training results

**Figure 9:**
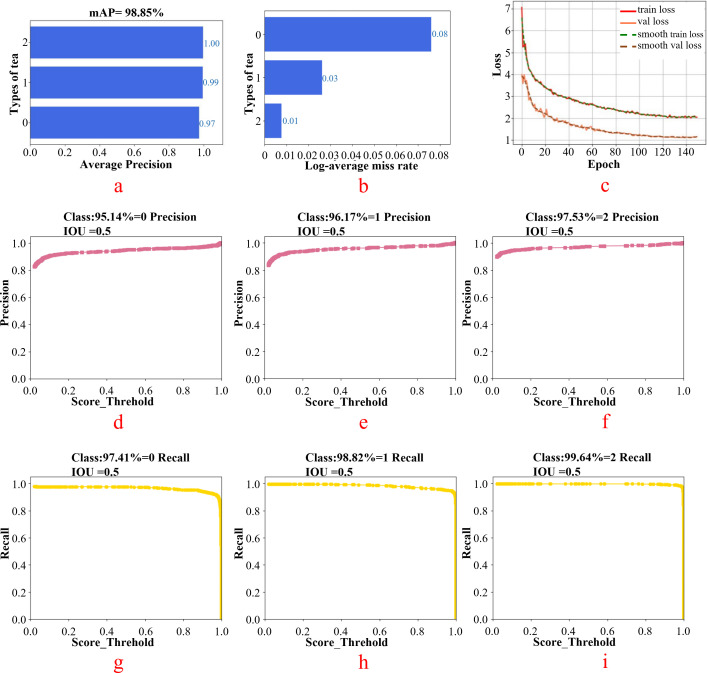
Faster R-CNN model test results [**(a)** tea average accuracy; **(b)** tea log _ average miss rate; (**(c–e)**: faccuracy of different grades of white tea at IOU = 0.5 ; **(g–i)**: the recall rate of different grades of white tea at IOU = 0.5].

The white tea grade data set was imported into the Faster R-CNN model for training. For the small target detection of white tea in this study, the batch size was set to nine. The results obtained after training are shown in **Figure 9**. **Figure 9A** shows the average accuracy (MAP) of different types of tea when the tea category is 0,1,2. **Figure 9B** shows the log _ average miss rate when the tea category is 0,1,2. The smaller the value, the better is the model. **Figure 9C** displays the training set loss function curve and validation set loss function curve. **Figures 9D–F** represent the accuracy of different grades of white tea at IOU = 0.5. **Figures 9G–I** present the recall rate of different grades of white tea at IOU = 0.5.

**Figure 10:**
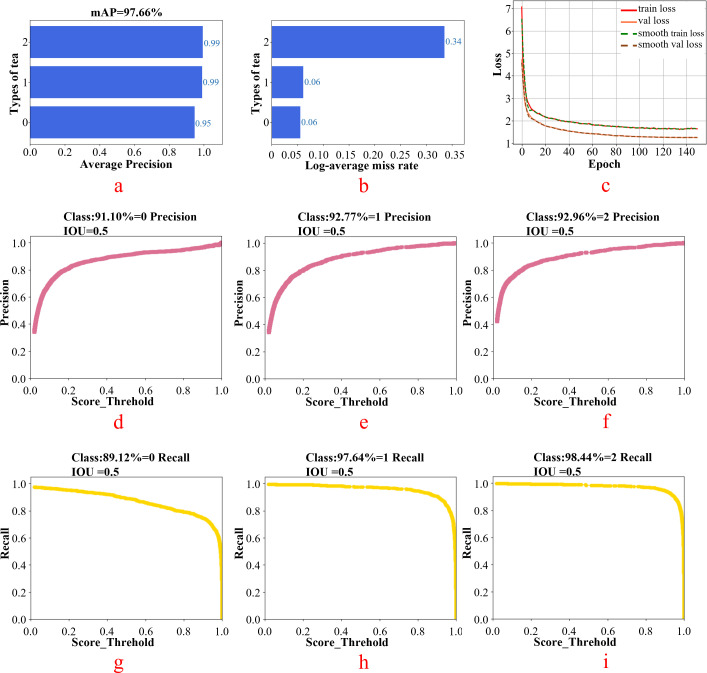
SSD model test results [**(a)** tea average accuracy; **(b)** tea log _ average miss rate; **(c–e)** faccuracy of different grades of white tea at IOU = 0.5 ; **(g–i)**: the recall rate of different grades of white tea at IOU = 0.5].

The white tea grade dataset was imported into the SSD model for training. The number of iterations in this experiment was 150. The batch size was set to eight for the small target detection of white tea in this study. The results obtained after training are shown in **Figure 10**. **Figure 10A** shows the average accuracy of the model to identify tea when the tea category is 0,1,2. **Figure 10B** shows the log _ average miss rate when the tea category is 0,1,2. The smaller the value, the better is the model training effect. **Figure 10C** displays the variation trend of the loss function curves of the training and validation sets with the increase in the number of training times. It can be observed from the figure that the model approaches a smooth state after 100 times of training. **Figures 9D–F** represent the accuracy of the model at IOU = 0.5 when the tea category is 0,1,2. **Figures 10G–I** present the recall rate of different grades of white tea at IOU = 0.5.

**Figure 11:**
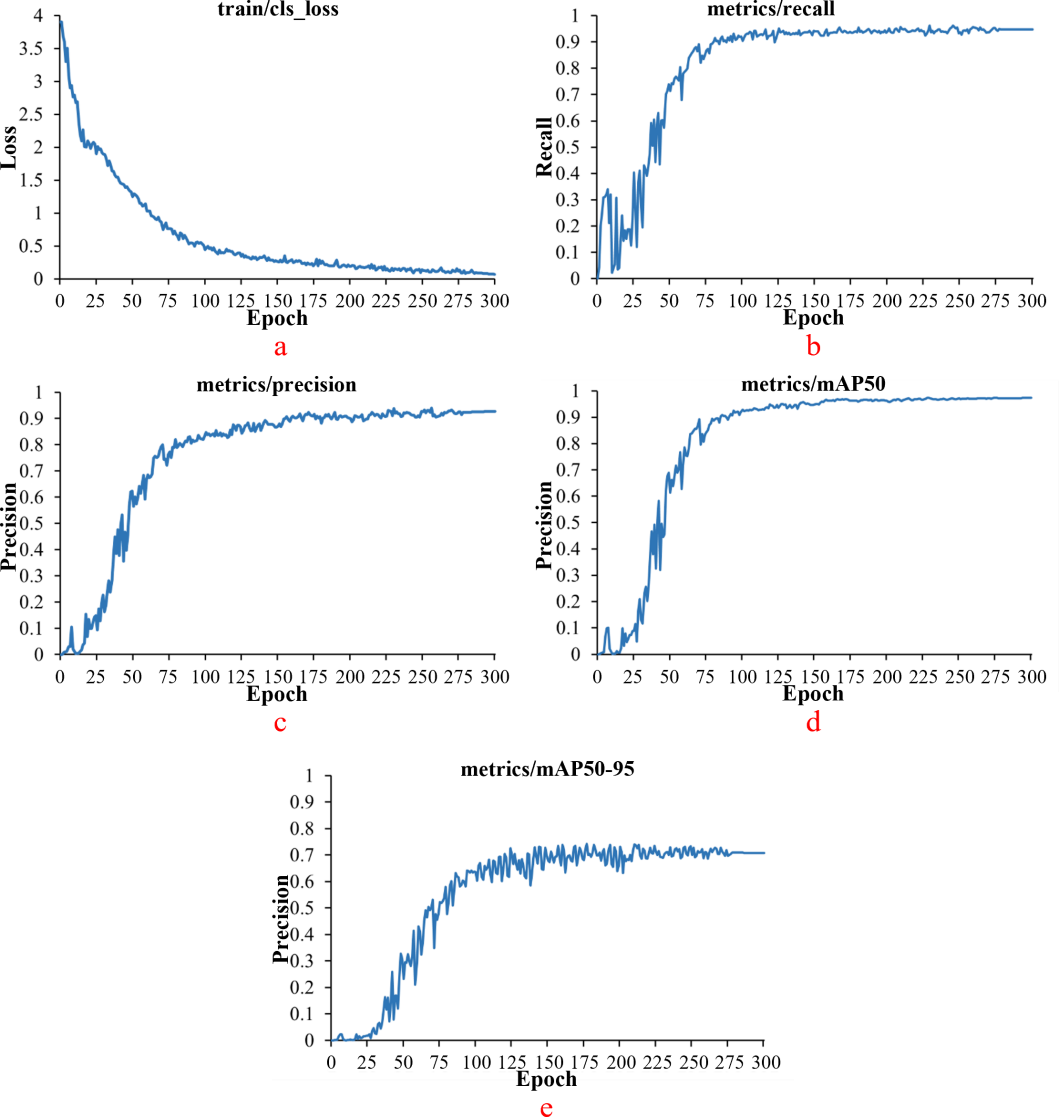
Experimental results of YOLOv11 model [**(a)** model cls loss; **(b)***model recall*; **(c)***model precision*; **(d)***model mAP* 0.5; **(e)***model mAP* _ 0.5 : 0.95].

The white tea grade dataset was imported into the YOLOV11 model for training. The number of iterations of this training was 300. Because this study was aimed at the tea target with a smaller target, the batch size was set to eight. After training, the experimental results of cls _ loss, recall, precision, MAP _ 0.5, MAP _ 0.5: 0.95 of the model are shown in **Figure 11**. After 75 iterations of model training, the curve is close to a stable trend. The cls _ loss of the model is close to 0.76. Precision approaches 0.77. The recall of the model approaches 0.86. The MAP _ 0.5 of the model is close to 0.84. The MAP _ 0.5: 0.95 of the model is close to 0.52.

**Figure 12:**
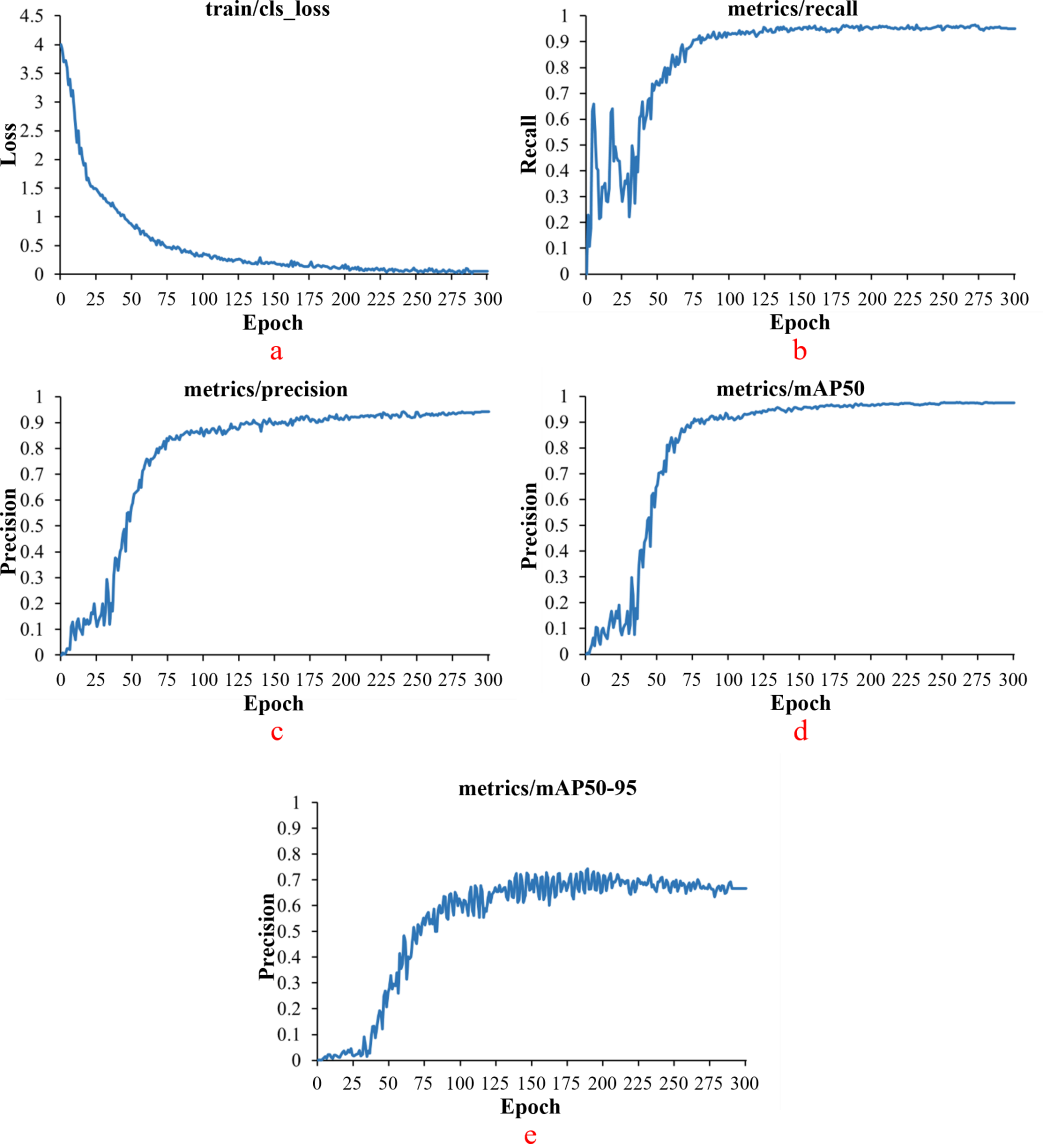
Experimental results for cls-loss in YOLO-AE model. [**(a)** model cls *loss*; **(b)***model recall*; **(c)***model precision*; **(d)***model mAP* 0.5; **(e)***model mAP* _ 0.5 : 0.95].

The white tea grade dataset was imported into the YOLO-AE model for training. The number of iterations of this training was 300. Because this study was aimed at the tea target with a smaller target, the batch size was set to eight. After training, the experimental results of cls _ loss, recall, precision, MAP _ 0.5, MAP _ 0.5: 0.95 of the model are shown in **Figure 12**. After 75 iterations of model training, the curve is close to a stable trend. The cls _ loss of the model is close to 0.47. Precision approaches 0.82. The recall of the model approaches 0.9. The MAP _ 0.5 of the model is close to 0.89. The mAP _ 0.5: 0.95 of the model is close to 0.52.

By training the four models, the corresponding model parameters were obtained. The test data of Precision, Recall, MAP _ 0.5, MAP _ 0.5: 0.95, and frame recognition time time for each model are shown in **Table 2**. The comparison results reveal that the weight file of the Faster R-CNN model is the largest. Moreover, these predict that an image requires the longest time. The SSD model displayed accuracy, recall, and MAP values lower than those for the other two models. Among the three models, the YOLO-AE model yielded the best training results. The model requires the shortest time to predict, the minimum weight file is 6.3 M, and the model is lightweight. In the identification, the shape parameters of different grades of tea are close, which requires high accuracy. The improved YOLOV11 model and the number of parameters and floating-point operations before the improvement only increased by 3%, but the recognition accuracy increased by 3%. Compared with the YOLOV5 model, the improved model parameters are reduced by 61%, and the recognition accuracy is increased by 3%. The accuracy of the system identification is the first pursuit factor. Based on the analysis of the above factors, this study chooses the improved YOLOV11 model to be more suitable for tea selection regulation.

**Table 2:** Statistical comparison of training results of three models.

Model	Category	Precision	Recall	mAP_0.5	mAP_0.5:0.95	Frame recognition time time/s
Faster R-CNN	0	94.14%	96.41%	98.65%	×	0.378
	1	95.07%	97.42%			
	2	96.43%	98.14%			
SSD	0	91.1%	88.02%	96.86%	×	0.242
	1	92.72%	96.61%			
	2	92.95%	97.34%			
YOLOv5s	0	93.3%	93.8%	92.21%	84.14%	0.086
	1	92.5%	91.4%			
	2	94.5%	93.5%			
YOLOv11	0	92.3%	92.2%	94.17%	91.64%	0.036
	1	94.5%	91.4%			
	2	93.7%	93.5%			
YOLO-AE	0	94.8%	95.9%	95.2%	91.7%	0.037
	1	95.4%	96.2%			
	2	96.2%	95.7%			

**Figure 13:**
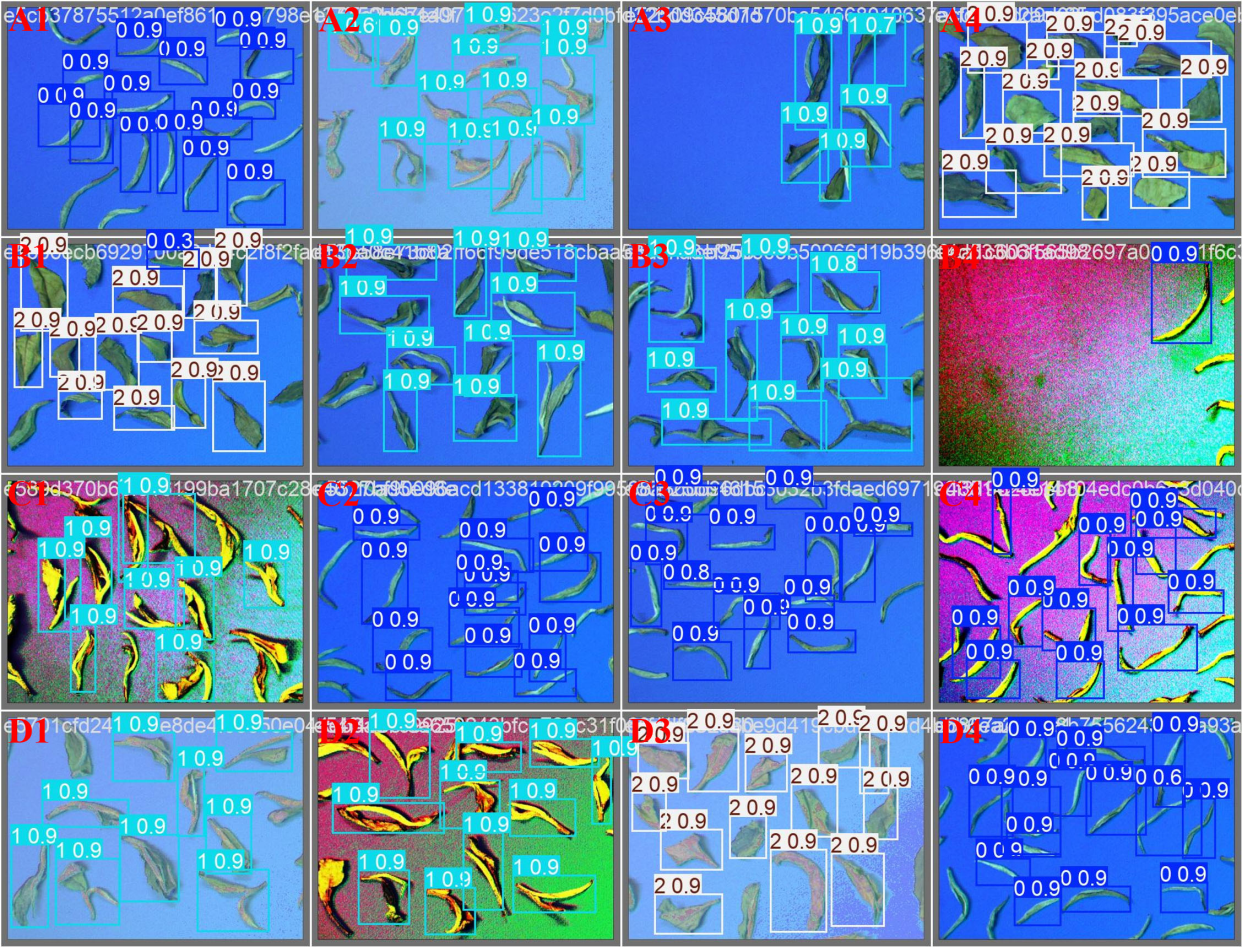
The verification result for tea grade recognition [**(A1, B4, C2, C3, C4, D4)** : category 0 recognition results ; **(A2, A3, B2, B3, C1, D1, D2)** : Category 1 recognition results ; **(A4, B1, D3)** : Category 2 recognition results].

Then, the experimental results were compared with those of Chuan et al (Yin et al., 2023). The recognition system in this study improved the overall recognition accuracy by an average of 3% through light adjustment, background plate selection, and dataset preprocessing.When tea leaves enter the recognition area, there is a small amount of overlap. In this study, tea diversion to avoid overlapping phenomenon. When the speed of tea entry is fast, the picture is blurred. The problem of image blurring is solved by increasing the frame rate of the camera. The tea part enters the camera field of view, resulting in misidentification. Based on the above factors, there are differences in the recognition accuracy of tea of the same grade. As shown in **Figure 13** B1. The probability of this occurrence in the actual test is less than 3%, so the impact on the final test results can be ignored.

### 3.2 Accuracy verification of YOLO-AE recognition system

The recognition accuracy of the recognition system based on the YOLO-AE model was verified. In this study, high-quality white tea in the white tea raw materials accounted for 85%, secondary white tea accounted for 10%, and broken leaf impurities accounted for 5% as test materials. The mixed tea was divided randomly into 10 parts. In turn, 10 tea leaves were put into the wind selection test bench for the identification test. The accuracy of the tea identification model was verified by 10 experiments. Finally, the feasibility of the system was analyzed based on the recognition time and accuracy. A set of recognition results is shown in **Figure 13**. The three tea categories were marked with three color border boxes. The number 0,1 and 2 on the left side of the label represent high-quality tea, secondary tea and. The values behind represent the recognition accuracy.There is a small amount of overlap in tea. In this study, tea diversion to avoid overlapping phenomenon. When the speed of tea entry is fast, the picture is blurred. The problem of image blurring is solved by increasing the frame rate of the camera. Incomplete tea leaves in the image lead to misidentification. Based on the above factors, there are differences in the recognition accuracy of tea of the same grade. As shown in **Figure 13**-B1. The probability of this occurrence in the actual test is less than 3%, so the impact on the final test results can be ignored.

The accuracy of the YOLO-AE model in identifying different types of tea was tested through 10 experiments. Finally, the statistical model recognition time, recognition accuracy of various grades of tea, and proportion of each component of tea were determined. The results are shown in **Table 3**. The results of the 10 experiments reveal that the recognition time for each image was less than 45 ms. The recognition accuracy for high-quality white tea, secondary white tea, and debris impurities was above 90%. The standard deviation of the recognition accuracy of the three kinds of tea is 9.73,9.72 and 9.75. The results show that the recognition accuracy is stable. The statistical analysis of the proportion of various teas was essentially consistent with the ratio set by oneself. The deviation of each tea proportion was ± 0.8%. Among these, the highest proportion of high-quality white tea raw materials had a better recognition effect (accounting for nearly 85%). Then, the experimental results were compared with those of Huang et al (Shaohua and Xifeng, 2022). The recognition accuracy of the recognition system in this study for various components of white tea materials was better than that of its improved model by 1–3%.

**Table 3:** Tea identification verification test.

Experimental group number	Recognition time (ms)	Accuracy for premium white tea (%)	Secondary white tea accuracy (%)	The accuracy rate for yellow flake impurities (%)	Proportion for tea of all grades (%)
1	41	94.13	94.22	96.78	85.4:9.1:5.5
2	42	95.35	93.97	95.66	84.3:9.5:6.2
3	43	96.19	91.33	95.21	84.1:10.1:5.8
4	37	95.83	94.01	95.88	84.4:9.5:6.1
5	38	96.11	94.89	92.12	84.4:9.3:6.3
6	36	92.12	96.11	92.13	85.5:9.1:5.4
7	41	96.82	94.24	96.89	84.3:10.5:5.2
8	41	96.01	95.02	94.56	85.8:9.1:5.1
9	42	92.34	94.33	97.21	85.3:8.5:6.2
10	38	92.34	97.05	94.88	85.2:9.3:5.5
standard deviation	6.32	9.73	9.72	9.75	×

### 3.3 Tea winnowing system experiment

To verify the separation effect of the air separation system, this study compared two proportions of white tea materials. The proportions of the first high-quality white tea, secondary white tea, and debris impurities were 85%, 10%, and 5%, respectively. The second high-quality white tea, secondary white tea, and debris impurities accounted for 70%, 22%, and 8%, respectively. The first batch of raw materials was fed first, followed by the second batch. Then, the variations in fan frequency and separator position were compared with the first batch of tea. In this study, 10 repeated experiments were performed. After the tea was identified by the recognition system, the wind selection parameters were adjusted, and the proportion of high-quality white tea at the discharge outlet was counted manually. The results are shown in **Table 4**.

**Table 4:** The second discharge port verification test.

Experimental group number	Fan wind speed difference (m/s)	Vibration feeder frequency difference (Hz)	The moving position difference of the separator (cm)	Difference in the proportion of high-quality tea (%)
1	1.8	11	2.5	1.3
2	2.7	11	3.1	1.6
3	1.5	11	1.6	2.1
4	1.7	11	1.4	0.7
5	1.8	11	2.2	1.1
6	1.1	11	2.8	1.3
7	2.5	11	1.7	1.5
8	2.2	11	2.5	1.1
9	1.9	11	1.3	1.2
10	1.3	11	1.6	0.6

The winnowing parameters required for the first tea and second tea differed significantly. The feeding speed remained unaltered. The maximum wind speed difference of the fan was 2.7 m/s, and the minimum was 1.1 m/s. The difference in the moving position of the partition plate was positive, and the partition plate moved in the same direction. The maximum difference was 3.1 cm, and the minimum difference was 1.3 cm. The maximum difference in the content of high-quality white tea at the second outlet was 2.1%, and the minimum value was 0.6%. The difference in the content of high-quality white tea was less than 3%. This result shows that the recognition system can adjust the wind selection parameters in real time with the change of tea materials for winnowing of different batches of tea.

In the actual tea production, the dim light, high noise and hot and humid environment lead to the inability to stay for a long time. Therefore, the wind selection parameters cannot be adjusted in time and accurately. At the same time, manual adjustment takes a long time. The system can quickly adjust the parameters in time.The tea identification environment is a closed space. The light is stabilized by an industrial light source and will not be affected by other light. As the use time increases, the dust of the tea will cover the lens, resulting in unclear pictures. In the subsequent use process, it is necessary to regularly clean the dust on the lens to ensure the stability of the shooting. At present, we are establishing a database of impurity sorting of green tea. Through the training of this model and the simulation of air separation parameters, the impurity separation of other kinds of tea can be realized.

## 4 Conclusion

This study addressed the problem wherein the air separation parameters of the conventional air separator cannot be adjusted in a timely manner according to different materials. Based on deep learning, a real-time adjustment system of wind selection parameters was designed. The recognition effects of the four recognition models were tested. Finally, it is found that the comprehensive parameters of the YOLO-AE recognition model are better than the other two models.A real-time wind selection parameter adjustment system was designed based on the previous research on wind selection parameters. The following conclusions were drawn:

The combination of wind separator and deep learning model can improve the quality and efficiency of wind separation. The real-time air separation adjustment system based on deep learning can adjust the air separation parameters in real time with the variation in the proportion of white tea materials. The system identification analysis required less than 2s. Combined with the previous study of winnowing parameters, the adjustment of winnowing machine parameters could be completed within 5s. Through practical experiments, the recognition accuracy and wind selection accuracy of high-quality tea satisfied the requirements of practical application.

The real-time statistics of the proportion of high-quality tea at the discharge outlet could be used to quantitatively analyze the accuracy of tea selection. Compared with the blind parameter adjustment of the conventional air separator, the accuracy of the real-time quantitative analysis of the discharge port can customize the proportion of tea materials. Additionally, it can satisfy the requirements of different types of tea.

Aiming at the requirement of high recognition accuracy for tea wind selection. The YOLO-AE recognition model has better adaptability in the accurate recognition of small target features of white tea materials. This study compares and analyzes the traditional recognition model, although it is similar to YOLO-AE in the number of model parameters. The recognition speed of the improved model is similar to that of the original model, but the accuracy is improved by 3%.

## Data Availability

The raw data supporting the conclusions of this article will be made available by the authors, without undue reservation.
